# Retinol Binding Protein 4 and Uric Acid as Risk Factors for Insulin Resistance in Type 2 Diabetes Mellitus

**DOI:** 10.1155/jdr/9446806

**Published:** 2026-06-08

**Authors:** Linyan Cheng, Liyan Wu, Tao-Hsin Tung, Zijun Chai, Yufen Zheng, Bo Shen

**Affiliations:** ^1^ Department of Clinical Laboratory, Taizhou Hospital of Zhejiang Province Affiliated to Wenzhou Medical University, Linhai, Zhejiang, China, wmu.edu.cn; ^2^ Key Laboratory of System Medicine and Precision Diagnosis and Treatment of Taizhou, Taizhou, Zhejiang, China; ^3^ Clinical Laboratory, Dongyang People′s Hospital, Dongyang, Zhejiang, China, dyhospital.com; ^4^ Evidence-Based Medicine Center, Taizhou Hospital of Zhejiang Province Affiliated to Wenzhou Medical University, Linhai, Zhejiang, China, wmu.edu.cn

**Keywords:** hyperuricemia, insulin resistance, retinol-binding protein 4, type 2 diabetes mellitus

## Abstract

**Background:**

Hyperuricemia (HUA) is closely associated with insulin resistance (IR). Retinol‐binding protein 4 (RBP4) plays a critical role in inducing IR. However, the combined effects of uric acid (UA) and RBP4 on IR remain unclear. This study aimed to identify the risk factors for IR in type 2 diabetes mellitus (T2DM) patients and to investigate the potential relationship between UA and RBP4 in this context.

**Methods:**

This study included 570 patients aged 18–60 years with T2DM who were treated at Taizhou Hospital in Zhejiang Province. Univariate and multivariate logistic regression analyses were performed to evaluate the associations of UA and RBP4 levels with IR risk in T2DM patients. Mediation analysis was conducted to assess the mediating role of RBP4 in the relationship between UA and homeostasis model assessment of insulin resistance (HOMA‐IR). Finally, ROC curve analysis was performed to examine the evaluation value of the model for IR in patients with T2DM.

**Results:**

RBP4 and UA levels were significantly correlated with HOMA‐IR (*p* < 0.01). Mediation analysis revealed that RBP4 partially mediated the relationship between UA and IR (*p* < 0.05, with the mediating effect accounting for 23.2%). Furthermore, both RBP4 and UA were identified as significant risk factors for IR in patients with T2DM (*p* < 0.05). The risk of IR associated with HUA was greater when RBP4 ≥ 36.6 mg/L (*p* < 0.001). The ROC analysis showed that compared with UA or RBP4 alone, the combination of RBP4 and UA was more effective in detecting IR and represented a superior assessment model (AUC = 0.788, *p* < 0.001).

**Conclusions:**

RBP4 played a partial mediating role between UA and IR. Both RBP4 and UA were risk factors for IR in T2DM patients. The combined use of these two indicators can better evaluate the risk of IR. Further research is needed to validate the reliability of using UA and RBP4 levels to evaluate IR.

## 1. Introduction

During social development, human lifestyles and dietary habits have become increasingly unhealthy, leading to a rise in metabolic diseases. Moreover, various metabolic diseases are closely interrelated. Patients often suffer from multiple conditions simultaneously, including diabetes, hyperuricemia (HUA), hyperlipidemia, hypertension, and obesity [1]. Type 2 diabetes mellitus (T2DM) is a chronic metabolic disorder that impairs carbohydrate metabolism [2]. Pancreatic *β*‐cell insulin resistance (IR) and impaired insulin secretion are central pathogenic mechanisms of T2DM and represent key therapeutic targets; these defects further contribute to systemic metabolic dysregulation of carbohydrates, lipids, and proteins [3]. HUA results from purine metabolism disorders and impaired serum uric acid (UA) excretion. This also represents a significant risk factor for T2DM [4]. Hyperinsulinemia and IR are frequently accompanied by elevated serum urate levels, which arise from the combined effects of genetic and environmental factors [5–8]. When diabetic nephropathy occurs, insufficient UA excretion further exacerbates HUA. Elevated UA levels are associated with a higher likelihood of developing T2DM, and HUA may contribute to the progression or aggravation of T2DM and its associated complications via multiple mechanisms [9, 10]. Studies have shown that the predictive effect of UA varies among patients with different degrees of IR [11]. Early intervention in patients with high IR levels can effectively alleviate the treatment burden associated with HUA and T2DM, and vice versa. These findings suggest that HUA is closely related to disorders of glucose metabolism and IR. However, the molecular mechanisms underlying IR remain unclear. Clarifying the role of HUA in IR is of great significance for the effective prevention and treatment of IR and T2DM.

As a member of the lipid transport protein family, retinol‐binding protein 4 (RBP4) exhibits high selectivity and affinity for vitamin A metabolites, known as retinoids, and is predominantly expressed in the liver and adipose tissue [12]. In addition, RBP4 functions as an adipokine that participates in the regulation of glucose metabolism and IR in skeletal and cardiac muscles, which may be linked to the development of metabolic syndrome [13]. A strong association between serum RBP4 and T2DM as well as IR has also been demonstrated [14]. Both clinical and basic studies have shown that serum RBP4 levels are positively correlated with T2DM and negatively correlated with islet function [15, 16]. Furthermore, research conducted by Zhou et al. revealed that RBP4 levels are positively associated with the homeostasis model assessment of insulin resistance (HOMA‐IR) and 2 h postprandial blood glucose levels in individuals with T2DM, suggesting its potential as a metabolic biomarker for evaluating pancreatic *β*‐cell function [17]. RBP4 can induce IR, increase the risk of T2DM, and inhibit insulin signaling through proinflammatory effects [18].

The research conducted by Wang et al. indicated that reducing the level of RBP4 may have contributed to the improvement of IR in obese patients [19]. Martínez‐Sánchez et al. reported that the UA concentrations are significantly correlated with IR and IR‐related disorders [10]. Current research mainly focuses on the relationship between UA or RBP4 and IR, but no study has examined the relationship between the two in combination with IR. HUA induces IR both in vivo and in vitro. Given the shared influence of RBP4 and UA on IR, it is speculated that RBP4 and UA may play a synergistic role in IR in patients with T2DM. Consequently, this study focused on investigating the risk factors related to IR in patients with T2DM and examining the relationship between UA and RBP4 in IR, thereby providing clues and evidence for the possible mechanism of IR in T2DM patients.

## 2. Methods

### 2.1. Research Population and Design

**Figure 1:**
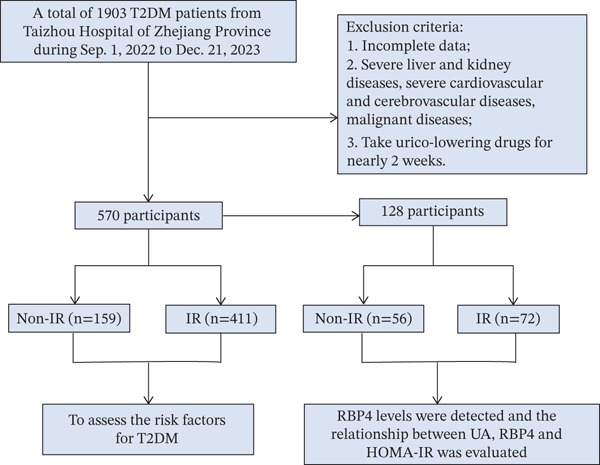
Flowchart of the participants selection process.

This study retrospectively collected clinical and laboratory data from 1903 patients aged 18–60 years with T2DM who were treated at Taizhou Hospital in Zhejiang Province between September 1, 2022, and December 21, 2023. Ultimately, 570 patients were included in the study (Figure 1). Fasting serum samples from 128 T2DM patients were randomly selected to measure RBP4 level. T2DM was diagnosed according to the relevant guidelines [20]. The exclusion criteria were as follows: (1) incomplete data; (2) acute cerebrovascular disease, severe renal insufficiency (estimated glomerular filtration rate (eGFR) < 30 mL/min/1.73 m^2^), or overt liver disease; (3) history of major cardiovascular diseases, malignant tumors, or immune‐related diseases; and (4) recent use of UA‐lowering drugs. The study was approved by the Ethics Committee of Taizhou Hospital of Zhejiang Province (Approval Number: K20220625), and all participants provided written informed consent before enrollment.

### 2.2. Data Collection

Standardized questionnaires were administered using face to face interviews to collect information on patients′ histories of smoking, drinking, hypertension, hyperlipidemia, and cardiovascular disease. A smoker was defined as an individual who consumed at least one cigarette per day for a duration of more than 6 months. A drinker was defined as an individual who consumed any type of alcohol at least once per week for a period of more than 6 months. General clinical data collected included sex, age, height, weight, body mass index (BMI), systolic blood pressure (SBP), and diastolic blood pressure (DBP). Laboratory indicators measured included UA, eGFR, triglycerides (TG), total cholesterol (TC), high‐density lipoprotein cholesterol (HDL‐C), low‐density lipoprotein cholesterol (LDL‐C), glycosylated hemoglobin (HbA1c), fasting blood glucose (FPG), and fasting serum insulin (FINS), all of which were obtained from the medical records of Taizhou Hospital of Zhejiang Province. The formula for calculating HOMA‐IR was as follows: HOMA − IR = FPG (mmol/L) × FINS (mIU/L)/22.5. A HOMA − IR value ≥ 2.69 was defined as IR [21].

### 2.3. Detection of RBP4

The concentration of RBP4 in blood was determined using an enzyme‐linked immunosorbent assay (ELISA) kit. Briefly, 50 *μ*L of standards and samples at different concentrations were added to each well, followed by the addition of 100 *μ*L of HRP‐conjugated antibody. The plate was then incubated at 37°C for 60 min. After incubation, the plate was washed five times with the prepared washing solution. Subsequently, 50 *μ*L of substrate A and 50 *μ*L of substrate B were added to each well of the plate. The plate was incubated at 37°C in the dark for 15 min. Subsequently, a stop solution was added, and the absorbance was immediately measured at 450 nm using a microplate reader. Finally, the RBP4 concentration in the samples was calculated based on the obtained readings.

### 2.4. Mediation Analysis

Causal mediation analysis was performed using the “mediation” package in R statistics (version 4.3.3) to estimate the mediation effect. The nonparametric bootstrap method (with 2000 resamples) was adopted to calculate the average causal mediation effect (ACME, i.e., indirect effect a × b), average direct effect (ADE, i.e., *c* ^′^), total effect, and proportion mediated. Specifically, UA was used as predictor variable (*X*), RBP4 was used as mediator (*M*), and the degree of IR in T2DM patients was used as the outcome variable (*Y*). In this framework, the “total effect” includes the “direct effect” (not mediated by *M*) and the “indirect effect” (fully or partially mediated by *M*). Sensitivity analysis using the residual correlation coefficient method was conducted to assess the robustness of the mediating effect, implemented with the medsens function in the mediation package of R.

### 2.5. Statistical Analysis

The participants were categorized into non‐IR and IR groups. For baseline characteristics, categorical variables were analyzed using the chi‐square test, normally distributed continuous variables using the independent *t*‐test, and nonnormally distributed continuous variables using the Mann–Whitney *U* test. Univariate and multivariate binary logistic regression analyses were performed to identify risk factors associated with IR. Pearson′s correlation test was used to assess the relationship between variables. ROC curve analysis was performed to evaluate the ability of the model to identify the development of IR in T2DM patients. All statistical analyses were performed using R statistics (version 4.3.3) and SPSS, with a *p* − value < 0.05 considered statistically significant.

## 3. Results

### 3.1. Characteristics of the Participants

To analyze the risk factors for T2DM, we measured several key metabolic indicators in T2DM patients. T2DM patients were classified into non‐IR and IR groups according to their HOMA‐IR levels. The baseline characteristics of the two groups are presented in Table 1. Compared with the non‐IR group, the IR group had a significantly higher prevalence of alcohol consumption, hypertension, and hyperlipidemia (*p* < 0.05). Regarding laboratory indicators, the IR group had markedly elevated levels of BMI, SBP, DBP, UA, TG, TC, HbA1c, FPG, and FINS compared with the non‐IR group, along with a lower level of HDL‐C (*p* < 0.01).

**Table 1:** Characteristics of the study population.

Characteristics	Overall (*n* = 570)	Non‐IR (*n* = 159)	IR (*n* = 411)	*p*
Age (years)	51.00 (42.00, 56.00)	51.00 (46.00, 56.00)	50.00 (41.00, 56.00)	0.389
Gender (Male), *n* (%)	361 (63.3)	103 (64.8)	258 (62.8)	0.727
Smoker, *n* (%)	178 (31.2)	54 (34.0)	124 (30.2)	0.438
Drinker, *n* (%)	98 (17.2)	36 (22.6)	62 (15.1)	0.043
Hypertension, *n* (%)	174 (30.5)	36 (22.6)	138 (33.6)	0.015
Hyperlipidemia, *n* (%)	215 (37.7)	46 (28.9)	169 (41.1)	0.009
Cardiovascular diseases, *n* (%)	26 (4.6)	11 (6.9)	15 (3.6)	0.146
BMI (kg/m^2^)	24.84 (22.56, 27.48)	23.81 (21.59, 25.64)	25.32 (22.84, 28.08)	< 0.001
SBP (mmHg)	130.00 (120.0, 140.00)	125.00 (119.00, 137.00)	131.00 (121.00, 142.00)	0.002
DBP (mmHg)	81.00 (72.25, 88.00)	78.00 (70.00, 85.00)	82.00 (73.00, 90.00)	< 0.001
UA (*μ*mol/L)	328.50 (269.0, 409.75)	305.00 (261.50, 355.00)	349.00 (275.0, 434.00)	< 0.001
eGFR (mL/min/1.73m^2^)	107.00 (99.00, 115.00)	105.00 (98.00, 113.00)	108.00 (99.00, 116.00)	0.085
TG (mmol/L)	1.55 (1.03, 2.43)	1.31 (0.89, 1.89)	1.67 (1.06, 2.76)	< 0.001
TC (mmol/L)	4.96 (4.25, 5.78)	4.75 (4.07, 5.51)	5.08 (4.30, 5.90)	0.009
HDL‐C (mmol/L)	1.12 (0.96, 1.33)	1.20 (1.02, 1.44)	1.10 (0.95, 1.30)	< 0.001
LDL‐C (mmol/L)	2.77(2.34, 3.34)	2.67 (2.28, 3.24)	2.84 (2.38, 3.34)	0.11
HbA1c (%)	10.20 (8.22, 11.80)	9.00 (7.05, 10.80)	10.60 (8.90, 12.10)	< 0.001
FPG (mmol/L)	9.14 (6.89, 11.60)	7.37 (6.08, 9.70)	9.79 (7.56, 12.22)	< 0.001
FINS (mU/L)	10.20 (6.70, 13.78)	5.00 (3.70, 7.05)	12.00 (9.00, 15.20)	< 0.001
HOMA‐IR	3.92 (2.52, 5.54)	1.87 (1.39, 2.22)	4.73 (3.63, 6.52)	< 0.001

Continuous data are presented as mean (standard deviation) or median (interquartile range), and categorical variables are presented as frequency (percentage).

BMI, body mass index; SBP, systolic blood pressure; DBP, diastolic blood pressure; UA, uric acid; eGFR, estimated glomerular filtration rate; TG, triglycerides; TC, total cholesterol; HDL‐C, high‐density lipoprotein; LDL‐C, low‐density lipoprotein cholesterol; HbA1c, glycated hemoglobin; FBG, fasting blood glucose; FINS, fasting insulin; HOMA‐IR, homeostasis model assessment of insulin resistance.

### 3.2. Risk Factors for IR in Patients With T2DM

**Figure 2:**
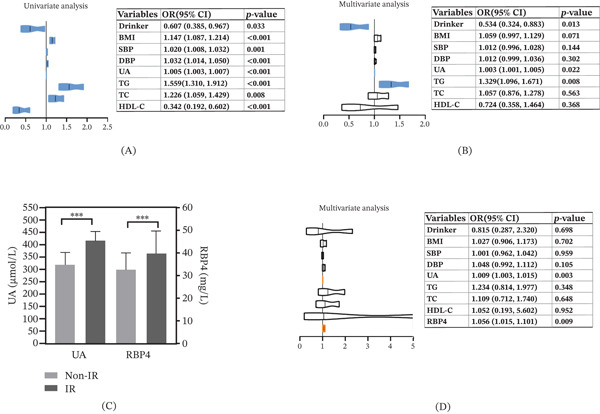
Logistic regression analysis of IR in T2DM patients: (A) Univariate logistic regression analysis of IR in 570 T2DM patients; (B) multivariate logistic regression analysis of IR in 570 T2DM patients; (C) UA and RBP4 levels in 128 T2DM patients; (D) multivariate logistic regression analysis of 128 T2DM patients after addition of RBP4.

Variables with *p* < 0.05 were incorporated into the univariate and multivariate regression analyses. Univariate regression analysis revealed that drinker, BMI, SBP, DBP, UA, TG, TC, and HDL‐C were all associated with the risk of IR in T2DM patients (*p* < 0.05), whereas only drinker, UA, and TG remained significant in the multivariate regression analysis (Figure 2A,B, *p* < 0.05). RBP4 levels were measured in 128 patients. Compared with the non‐IR group, both UA and RBP4 levels were significantly elevated in the IR group (Figure 2C, *p* < 0.001). After including RBP4 in the multivariate regression analysis, only UA and RBP4 were significantly associated with the risk of IR (Figure 2D, *p* < 0.05). Comparison of baseline characteristics between the main cohort and the RBP4 measurement cohort revealed no significant differences (*p* > 0.05). Thus, no selection bias was present in the baseline characteristics (Table S1).

### 3.3. Relationship Between UA, RBP4, and HOMA‐IR in Patients With T2DM

**Figure 3:**
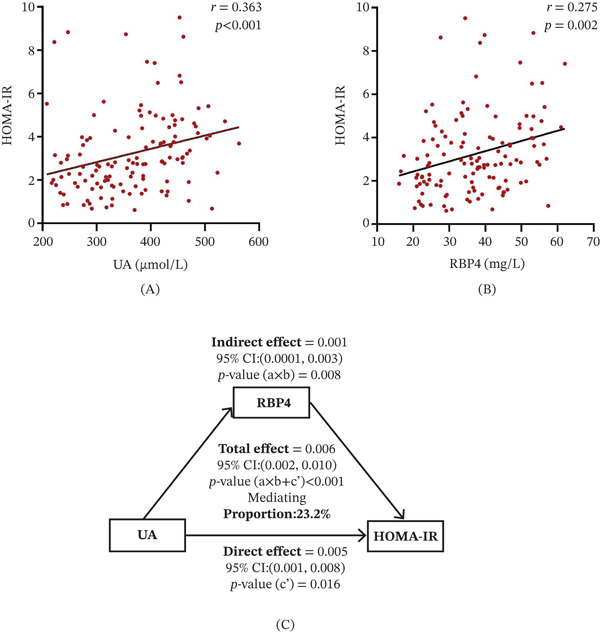
Correlation between UA, RBP4 and HOMA‐IR in T2DM patients. (A) The correlation between UA and HOMA‐IR; (B) the correlation between RBP4 and HOMA‐IR. (C) Mediation analysis of RBP4 between UA and HOMA‐IR. Adjusted for drinker, BMI, SBP, DBP, TG, TC, and HDL‐C.

Spearman correlation analysis revealed positive correlations between UA and HOMA‐IR and between RBP4 and HOMA‐IR (Figure 3A and B, *p* < 0.01). Of these, UA showed a stronger correlation with HOMA‐IR (*r* = 0.363, *p* < 0.01). A mediation analysis was performed with UA as the predictor variable (*X*), RBP4 as the mediator (*M*), and IR in T2DM patients as the outcome variable (*Y*). The findings revealed a direct association between UA and HOMA‐IR [*p*(c^′^) < 0.05], and UA had a potential causal effect on HOMA‐IR mediated by RBP4 [*p*(a × b) < 0.05]. RBP4 exerted a partial mediating role in the relationship between UA and HOMA‐IR, with a mediation effect ratio of 23.2% (Figure 3C). Sensitivity analysis of the indirect effect was conducted. The results showed that the robustness was weak, suggesting that this mediating pathway was susceptible to confounding factors (Figure S1).

### 3.4. Subgroup Analysis Was Performed to Assess the Association of UA, RBP4, With IR

Patients were stratified based on the presence or absence of HUA and RBP4 levels (< 36.6 or ≥ 36.6 mg/L) (Table 2). The findings indicated that HUA was independently linked to IR, regardless of adjustment (*p* < 0.05). This independent association was stronger in T2DM patients with both HUA and RBP4 ≥ 36.6 mg/L (*p* < 0.001).

**Table 2:** Subgroup analysis of the effects of HUA and RBP4 on the risk of IR.

	OR (95% CI)	*p*
Unadjusted		
Non‐HUA and RBP4 < 36.6 mg/L	Ref	
Non‐HUA and RBP4 ≥ 36.6 mg/L	1.872 (0.692, 5.192)	0.22
HUA and RBP4 < 36.6 mg/L	5.398 (1.879, 16.832)	0.002
HUA and RBP4 ≥ 36.6 mg/L	10.606 (3.611, 35.473)	< 0.001

Adjusted		

Non‐HUA and RBP4 < 36.6 mg/L	Ref	
Non‐HUA and RBP4 ≥ 36.6 mg/L	1.609 (0.549, 4.807)	0.387
HUA and RBP4 <36.6 mg/L	5.018(1.481, 18.674)	0.012
HUA and RBP4 ≥ 36.6 mg/L	9.931(2.906, 38.953)	< 0.001

Adjusted for drinker, BMI, SBP, DBP, TG, TC, and HDL‐C. The median RBP4 concentration was divided into < 36.6 and ≥ 36.6 mg/L.

### 3.5. Predictive Value of UA and RBP4 for IR

**Figure 4:**
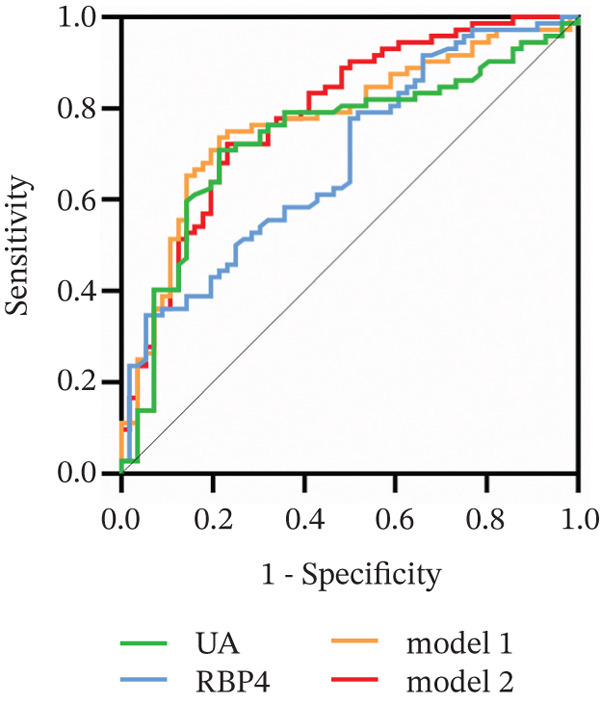
ROC curves of UA and RBP4 for predicting IR in T2DM patients. Model 1: UA and RBP4; Model 2: UA, RBP4, drinker, BMI, SBP, DBP, TG, TC, and HDL‐C.

The AUC of UA for assessing IR risk in T2DM patients was 0.737 (*p* < 0.001), and the AUC of RBP4 for assessing the same risk was 0.686 (*p* < 0.001). The best evaluation model performance was achieved by model 2, which had an AUC of 0.788 (*p* < 0.001). This model incorporated both UA and RBP4 and was adjusted for drinker, BMI, SBP, DBP, TG, TC and HDL‐C. These results suggest that the combination of UA and RBP4 has higher evaluation value than either UA or RBP4 alone (Table 3 and Figure 4). The probability formula for assessing IR is as follows:

Logit IR=−10.390.0088170.054144+×UA μmol/L+×RBP mg/L  +0.0010210.04689×SBP mmHg+×DBP mmHg  +0.025060.2099×BMI kg/m2+×TG mmol/L  +0.10330.05087×TC mmol/L+×HDL−C mmol/L  +−0.2048×Drinker yes=10/no=.

**Table 3:** The value of UA and RBP4 in predicting the risk of IR in T2DM patients.

Variables	AUC (95% CI)	Cut‐off	Sensitivity	Specificity	Youden index	*p*
UA	0.737(0.647, 0.827)	373	0.708	0.786	0.494	< 0.001
RBP4	0.686(0.595, 0.778)	46.43	0.347	0.946	0.293	< 0.001
Model 1	0.774(0.691, 0.856)	0.574	0.736	0.786	0.522	< 0.001
Model 2	0.788(0.709,0.867)	0.575	0.722	0.768	0.490	< 0.001

Model 1: UA and RBP4;

Model 2: Drinker, UA, RBP4, BMI, SBP, DBP, TG, TC, and HDL‐C.

## 4. Discussion

This study aimed to explore the risk factors for IR in T2DM patients and found that both RBP4 and UA were significantly associated with IR. Mediation analysis results revealed that RBP4 may partially mediate the relationship between UA and IR. More importantly, the combined levels of RBP4 and UA emerged as a notable risk factor for IR in T2DM patients. Notably, ROC analysis further indicated that the combination of RBP4 and UA is more effective in assessing IR than either marker alone and represents a superior evaluation model.

A growing body of research has shown that HUA is strongly linked to abnormal glucose metabolism and IR. IR assessment indicators (HOMA‐IR, TyG, and TG/HDL‐C) are closely associated with HUA. Patients with IR have higher UA levels, which are related to an increased risk of T2DM [22–25]. HUA may induce a significant increase in IR by suppressing the production of endothelial nitric oxide and increasing intracellular ROS levels, thereby increasing the risk of T2DM [26]. In both the liver and adipose tissue, elevated UA levels lead to higher oxidative stress and production of reactive oxygen species (ROS), which in turn triggers IR via the IRS1/Akt signaling pathway [27]. Consistent with these studies, the present study found that UA and RBP4 were the only factors significantly associated with the risk of IR in T2DM patients. Correlation analysis revealed a positive relationship between UA and HOMA‐IR. These results indicate that UA may increase the likelihood of IR in the T2DM population and serve as an independent risk factor for IR.

In addition, the nonclassical functions of RBP4, including embryonic development, insulin sensitivity, metabolism, and cardiovascular diseases have been extensively studied in recent years [12, 28]. RBP4 is reportedly associated with various risk factors for metabolic diseases, including hyperlipidemia, HUA, obesity, IR, and T2DM [17, 29, 30]. Among them, increased serum RBP4 levels can contribute to the development of IR and play a role in the progression of T2DM [31]. Kovacs et al. revealed that serum RBP4 levels were positively correlated with adipose RBP4 levels and intra‐abdominal fat mass but inversely correlated with insulin sensitivity [32]. RBP4 may activate the NLRP3 inflammasome via the TLR4/MD2 receptor complex and TLR2, thereby disrupting insulin signaling in adipocytes [18]. The study by Mazidi et al. indicated that visceral fat tissue completely mediates the relationship between UA and glucose/insulin homeostasis parameters, and RBP4, as a visceral fat factor, may be involved in this process [33]. This study further demonstrated that, similar to UA, RBP4 is a risk factor for IR and is positively correlated with the likelihood of IR development. T2DM patients may reduce IR levels by maintaining RBP4 levels below 36.6 mg/L.

Given that both UA and RBP4 are closely related to IR, but the relationships among these three factors have not been clarified, we conducted a mediation analysis on UA, RBP4, and HOMA‐IR and found that IR is partially mediated by RBP4, indicating that an indirect pathway of UA → RBP4 → IR exists in patients with T2DM. It is worth noting that the proportion of the indirect effect (23.2%) reflects the statistical result between UA and RBP4 in a single cross‐sectional dataset (assuming no undetected confounding factors). Sensitivity analysis also showed that even with moderate unmeasured confounding factors, the estimated indirect effect may be offset. Current literature indicates that insulin resistance is associated with age, genetics, obesity, HUA, hyperlipidemia, cardiovascular disease, inflammation, polycystic ovary syndrome, and so forth. [34]. The indicators measured in this study cover most of the possible confounding factors reported in most studies, and the influence of undetected variables on IR is usually limited. The mechanisms involved in the causal relationship between hyperuricemia and IR are still under investigation and have not been fully clarified [35, 36]. In terms of insulin signal regulation, both high UA and RBP4 levels can cause IR by inhibiting the IRS/AKT signaling pathway [37–39]. In an animal study utilizing HUA rat models, after RBP4 expression was suppressed, the phosphorylation levels of the IRS/PI3K/Akt signaling pathway were substantially increased, and IR in HUA rats was markedly improved [40]. In terms of inducing inflammation, both UA and RBP4 can activate the NF‐*κ*B signaling pathway, promoting the release of proinflammatory cytokines. These cytokines interfere with the insulin signaling pathway through a bypass mechanism, thereby inducing IR [41, 42]. Therefore, UA and RBP4 do not merely independently cause IR; rather, there is a close synergistic relationship between them. The combination of the two factors assessed IR risk better, especially in T2DM patients with HUA and RBP4 ≥ 36.6 mg/L. We also developed an evaluation model (Model 2). After adjustment, the AUC of UA and RBP4 in assessing IR was 0.788, which was greater than that of either UA or RBP4 alone. When the assessed probability of IR exceeds 0.575, IR could be determined (sensitivity = 72.2*%*, specificity = 76.8*%*). UA is a biomarker for identifying hyperuricemia, IR, chronic kidney disease, and the risk of cardiovascular metabolic diseases. RBP4 promotes de novo lipogenesis and inflammation in hepatocytes while interfering with insulin signaling. Combining UA with inflammation‐related indicators such as RBP4 may enable at‐risk patients to assess the possibility of IR occurrence in advance, as well as the occurrence of other metabolic diseases, obesity, and inflammation [43–45].

This study had several limitations. First, as this was a single‐center study, a key limitation was the relatively small sample size, which may have introduced selection bias and thereby limited the generalizability of the findings. Therefore, future research should prioritize large‐scale multicenter studies to validate and extend these results. Additionally, given the retrospective design, it was challenging to fully account for all possible confounding factors, and the underlying mechanisms remain incompletely understood. Future research should expand the target population to include healthy individuals and individuals of other ethnicities. Furthermore, we were interested in the factors influencing UA and RBP4 levels. Therefore, further research is necessary to clarify the relationships and molecular mechanisms linking UA, RBP4, and IR.

## 5. Conclusion

This study demonstrated that UA and RBP4 levels were significantly and positively associated with IR risk in patients with T2DM. Notably, the association between UA and IR was stronger. Furthermore, HUA exerted a greater influence on the risk of IR in T2DM patients when RBP4 levels were ≥ 36.6 mg/L. Mediation analysis indicated that RBP4 levels partially mediated the relationship between UA and IR. UA and RBP4 have good evaluation value for IR levels in T2DM patients, and the combined use of these two indicators provides the best assessment performance. In the future, more studies are needed to verify the reliability of using UA and RBP4 to evaluate IR. Furthermore, it is necessary to validate whether the increase in UA precedes the increase in RBP4 in T2DM patients through repeated measurements or prospective cohort studies.

## Author Contributions

**Linyan Cheng:** writing – review and editing, writing – original draft, visualization, formal analysis, conceptualization. **Liyan Wu:** writing – review and draft, data curation, methodology, conceptualization, investigation. **Tao-Hsin Tung:** supervision, writing – review and editing, software. **Zijun Chai:** investigation, data curation. **Yufen Zheng:** writing – review and editing, supervision, formal analysis, funding acquisition. **Bo Shen:** writing – review and editing, formal analysis, supervision, conceptualization, project administration. Linyan Cheng and Liyan Wu have contributed to the work equally and should be regarded as co‐first authors.

## Funding

This study was supported by the Science and Technology Plan Project of Taizhou, (10.13039/501100018552, 25ywa11) and Scientific Research Foundation of Taizhou Enze Medical Center (Group) (2025EZZD05).

## Ethics Statement

The study received ethical approval from the Ethics Committee of Taizhou Hospital of Zhejiang Province (Approval Number: K20220625). All procedures were conducted in accordance with the ethical standards of the institution and the Declaration of Helsinki.

## Conflicts of Interest

The authors declare no conflicts of interest.

## Supporting information


**Supporting Information 1** Additional supporting information can be found online in the Supporting Information section. Table S1: Comparison of baseline characteristics between the main and the RBP4 detection cohorts. Figure S1: Sensitivity analysis of average causal mediation effect (ACME) vs. residual correlation (*ρ*). When *ρ* = 0.2 (0.05 to 0.35), it intersects with the ordinate at 0.

## Data Availability

The datasets used during the current study are available from the corresponding author on reasonable request.
