# Supplementary material for: Retinol Binding Protein 4 and Uric Acid as Risk Factors for Insulin Resistance in Type 2 Diabetes Mellitus
Source: J Diabetes Res. 2026 Jun 8;2026:9446806. doi: 10.1155/jdr/9446806 (PMC13244560; doi:10.1155/jdr/9446806)
Supplement: Supplementary file 1 — Supporting Information 1 Additional supporting information can be found online in the Supporting Information section. Table S1: Comparison of baseline characteristics between the main and the RBP4 detection cohorts. Figure S1: Sensitivity analysis of average causal mediation effect (ACME) vs. residual correlation (ρ). When ρ = 0.2 (0.05 to 0.35), it intersects with the ordinate at 0. [file JDR-2026-9446806-s001.docx]

**Supplementary Table 1** Comparison of baseline characteristics between the main and the RBP4 detection cohorts

| **Characteristics** | **Overall**  **(n=570)** | **RBP4 detection Cohort (n=128)** | ***p*-value** |
| --- | --- | --- | --- |
| Age (years) | 48.54 (9.55) | 47.67 (8.99) | 0.351 |
| Gender(Female), n (%) | 209 (36.7) | 59 (46.1) | 0.060 |
| Smoker, n (%) | 178 (31.2) | 35 (27.3) | 0.450 |
| Drinker, n (%) | 98 (17.2) | 26 (20.3) | 0.480 |
| Hypertension, n (%) | 174 (30.5) | 34 (26.6) | 0.436 |
| Hyperlipidemia, n (%) | 215 (37.7) | 38 (29.7) | 0.108 |
| Cardiovasculardiseae, n (%) | 26 ( 4.6) | 5 ( 3.9) | 0.930 |
| BMI (kg/m^2^) | 25.15 (3.83) | 25.49 (3.68) | 0.354 |

Continuous data are presented as the mean (standard deviation), while categorical variables are presented as frequency (percentage).


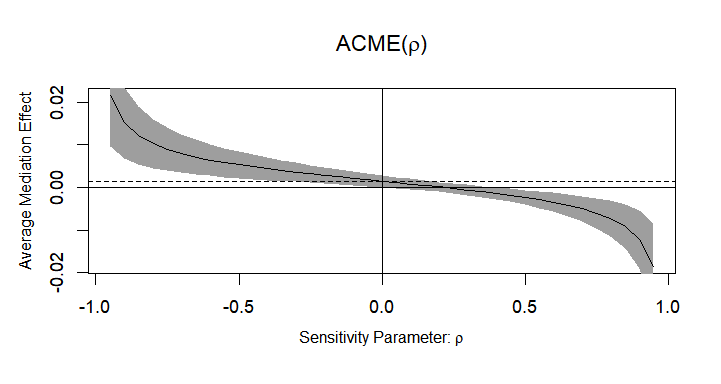


**Supplementary Figure 1** Sensitivity Analysis of Average Causal Mediation Effect (ACME) vs. Residual Correlation (ρ). When ρ=0.2 (0.05 to 0.35), it intersects with the ordinate at 0.
